# Comparative Clinical Outcomes and Safety of Generic Versus Original Imatinib in the Treatment of Chronic Myeloid Leukemia: A Real-World Cohort Study from Thailand

**DOI:** 10.3390/jcm14113695

**Published:** 2025-05-25

**Authors:** Jirapath Tangkitchot, Adisak Tantiworawit, Piangrawee Niprapan, Nuttanun Wongsarikan, Sirichai Srichairatanakool, Teerachat Punnachet, Nonthakorn Hantrakun, Pokpong Piriyakhuntorn, Thanawat Rattanathammethee, Chatree Chai-Adisaksopha, Ekarat Rattarittamrong, Lalita Norasetthada, Sasinee Hantrakool

**Affiliations:** 1 Department of Internal Medicine, Faculty of Medicine, Chiang Mai University, Chiang Mai 50200, Thailand; jirapath.t@cmu.ac.th (J.T.);; 2 Division of Hematology, Department of Internal Medicine, Faculty of Medicine, Chiang Mai University, Chiang Mai 50200, Thailand; adisak.tan@cmu.ac.th (A.T.);

**Keywords:** chronic myeloid leukemia, imatinib, generic drug, tyrosine kinase inhibitor, treatment outcome

## Abstract

**Background/Objectives**: Imatinib, a first-generation tyrosine kinase inhibitor, is the standard treatment for chronic myeloid leukemia (CML). Although generic formulations have improved access, concerns regarding their efficacy and safety remain. This study aimed to compare the clinical outcomes and adverse events of original and generic imatinib in patients with CML in Thailand. **Methods**: We conducted a retrospective cohort study of patients with chronic-phase CML receiving frontline imatinib at Chiang Mai University Hospital between January 2012 and September 2022. Treatment responses, event-free survival (EFS), overall survival (OS), and adverse events were also analyzed. **Results**: Among the 71 patients, 46 (64.8%) received original imatinib, and 25 (35.2%) received generic imatinib. The median follow-up period was 80.3 months (IQR: 52.0–106.4). At 12 months, there were no significant differences in the cumulative complete cytogenetic response (73.3% vs. 70.8%, *p* = 0.825) or major molecular response (35.6% vs. 41.7%, *p* = 0.618). Although EFS was not significantly different between the groups, OS was significantly longer in patients receiving original imatinib (*p* < 0.001). **Conclusions**: Although early treatment responses and EFS were similar, generic imatinib was associated with an inferior OS. These findings highlight the need for close monitoring and further evaluation of long-term outcomes when using generic formulations.

## 1. Introduction

Chronic myeloid leukemia (CML) is a clonal myeloproliferative neoplasm characterized by the presence of the Philadelphia (Ph) chromosome, resulting from a reciprocal translocation between chromosomes 9 and 22. This translocation leads to the formation of the BCR::ABL1 fusion gene, which encodes a constitutively active tyrosine kinase that promotes the uncontrolled proliferation of myeloid cells [1].

The landmark IRIS trial in 2003 transformed the standard of care in CML treatment by demonstrating the superiority of imatinib, a first-generation tyrosine kinase inhibitor (TKI), over the previous standard regimen of interferon-alpha and low-dose cytarabine in terms of hematologic and cytogenetic responses, tolerability, and disease progression [2]. Imatinib was approved by the United States Food and Drug Administration (U.S. FDA) in 2001 for the treatment of CML and other malignancies, such as gastrointestinal stromal tumors (GIST). Long-term follow-up of the IRIS study further confirmed the durability of responses and favorable overall survival (OS) outcomes with imatinib therapy, establishing it as the cornerstone of CML treatment [3].

Despite its efficacy, the high cost of original imatinib (Glivec^®^) poses a significant barrier to long-term treatment, especially in low- and middle-income countries. To address this issue, several generic formulations of imatinib have been introduced to improve access and reduce treatment costs while maintaining comparable efficacy and safety profiles [4,5,6,7,8,9,10,11,12].

Several international studies have evaluated the effectiveness of generic imatinib, with variable results [4,5,6,7,8,9,10,11,12]. While some studies have demonstrated comparable efficacy between original and generic formulations [5,6,7,8,11,12,13,14], others have raised concerns [15,16]. For instance, one study found that patients treated with generic imatinib were more likely to discontinue treatment due to lower persistence and increased intolerance [15]. Additionally, an observational study reported higher early failure rates in patients receiving generic imatinib, although this difference diminished over time [16]. In Southeast Asia, particularly Thailand, real-world data comparing the two formulations remain limited. This study aimed to evaluate and compare the clinical efficacy, treatment responses, long-term outcomes, and adverse event profiles of generic and original imatinib in patients with chronic-phase CML treated at a tertiary care center in Northern Thailand.

## 2. Materials and Methods

This retrospective cohort study with a historical control design was conducted at Chiang Mai University Hospital, Thailand. This study compared patients with CML who received original imatinib (diagnosed between 2012 and 2022) and those who received generic imatinib (diagnosed from 2017 onward) following a nationwide policy change in drug availability. This study was approved by the Institutional Review Board of the Faculty of Medicine, Chiang Mai University (Study number: MED-2564-08017; Research ID: 8017). The requirement for informed consent was waived due to the retrospective nature of the study. All patient data were fully anonymized prior to analysis, and confidentiality was strictly maintained in accordance with institutional policies and international ethical standards, including the Declaration of Helsinki.

The medical records of patients aged ≥15 years who were diagnosed with chronic-phase CML between January 2012 and September 2022 were reviewed. Eligible patients received imatinib, either the original or generic formulation, as first-line therapy. The diagnosis of CML was confirmed by the presence of the Ph chromosome or BCR::ABL1 fusion gene using conventional cytogenetics, fluorescence in situ hybridization (FISH), or polymerase chain reaction (PCR) techniques, in accordance with the 2013 European LeukemiaNet (ELN) criteria [17].

Demographic characteristics, baseline laboratory values, CML risk scores (Sokal and ELTS), Eastern Cooperative Oncology Group (ECOG) performance status, treatment responses, adverse events (AEs), treatment switching, and survival outcomes were collected from medical records.

For treatment allocation and drug formulation, all patients with CML in Thailand initially received original imatinib through a non-profit patient assistance program, regardless of their healthcare coverage. The program was discontinued in 2017. Thereafter, the type of imatinib prescribed depended on the patient’s healthcare plan. Patients under the Social Security Scheme (SSS) and Universal Coverage Scheme (UCS) were provided with generic imatinib, which was centrally procured by government agencies through periodic tenders. This may lead to variations in generic brands over time. Patients under the Civil Servant Medical Benefit Scheme (CSMBS) could receive either original or generic imatinib, depending on the clinical judgment of the treating physician and the policies or context of each center. In this retrospective cohort study, patients in the generic imatinib group were diagnosed from 2017 onward, following the national policy transition.

Treatment responses were classified according to the 2013 ELN recommendations as optimal, warning, or failure [17]. Cytogenetic and molecular responses were assessed using bone marrow cytogenetics and BCR::ABL1 transcript levels. Complete hematologic response (CHR), complete cytogenetic response (CCyR), and major molecular response (MMR) were recorded at 3, 6, and 12 months. Patients with optimal or warning responses continued with either treatment, while those with treatment failure were switched to second-generation TKIs.

Additional cytogenetic abnormalities (ACAs) were defined as any chromosomal abnormality detected in Ph chromosome-positive metaphases, except for the t(9;22) translocation. Major route abnormalities, as categorized by the ELN, include +8, +Ph, isochromosome 17q [i(17q)], +19, −7, and deletion 7q (del(7q)) [18].

The primary outcomes were the proportions of patients who achieved CCyR and MMR at 12 months. Secondary outcomes included response rates at 3 and 6 months, rates of treatment failure and switching, adverse events, event-free survival (EFS), and overall survival (OS). EFS was defined as the time from initiation of imatinib therapy to one of the following events: disease progression to accelerated phase (AP) or blastic phase (BP), loss of hematologic, cytogenetic, or molecular response, treatment discontinuation due to any cause, or death. OS was defined as the time from treatment initiation to death from any cause.

Statistical analysis was performed using descriptive statistics to summarize the patient characteristics. Continuous variables are reported as mean ± standard deviation (SD) or median (interquartile range, IQR), and categorical variables are reported as frequencies and percentages. Comparisons between groups were performed using the chi-square test or Fisher’s exact test for categorical variables and t-test or Mann-Whitney U test for continuous variables.

To evaluate trends in treatment response over time, we used a mixed-effects logistic regression model with random intercepts for each patient. This approach accounted for repeated measures of treatment response (e.g., CHR, CCyR, and MMR) at 3, 6, and 12 months. The primary variable of interest was the interaction between the treatment group (original vs. generic imatinib) and time. We assumed that the missing response data were missing at random, and no imputation was performed.

Survival outcomes (EFS and OS) were estimated using Kaplan-Meier analysis, with comparisons using the log-rank test. Cox proportional hazards regression models were used to assess the factors associated with EFS and OS. To account for differences in follow-up duration, a time-dependent Cox regression analysis was also performed. Variables with *p* < 0.05 in the univariable analysis were included in the multivariable models using a stepwise approach. Statistical analyses were performed using Stata version 16 (StataCorp LLC, College Station, TX, USA), and a two-tailed *p*-value of < 0.05 was considered statistically significant.

The sample size was estimated based on the previously reported CCyR rate of 79.1% in CML patients treated with imatinib at Chiang Mai University [19]. Using this as the expected proportion and allowing for a 95% confidence level with a margin of error of approximately 11%, the estimated sample size was approximately 70 patients. This was considered adequate for estimating the treatment response and allowed feasible enrollment for a real-world study. Additionally, the sample size was consistent with a comparative design, assuming a 20% difference in response rates between the original and generic imatinib, with 80% power and a 2:1 allocation ratio.

## 3. Results

### 3.1. Baseline Characteristics

A total of 71 patients with chronic-phase CML were included in the study. Among them, 46 (64.8%) received original imatinib, and 25 (35.2%) received generic imatinib as first-line treatment. The median age at diagnosis in the overall cohort was 40.0 years (IQR 33–54). Patients in the generic imatinib group tended to be older, with a median age of 50.0 years (IQR 33–60), compared to 38.5 years (IQR 33–49) in the original imatinib group; however, this difference was not statistically significant (*p* = 0.061). A total of 39 patients (54.9%) were male, with no significant difference in sex distribution between the groups.

Most patients had an ECOG performance status of 0 or 1. Sokal risk classification revealed that 28.2% were low-risk, 23.9% intermediate-risk, and 47.9% high-risk. ELTS scores categorized 42.2% of patients as low-risk, 28.2% as intermediate-risk, and 29.6% as high-risk. The mean hemoglobin level at diagnosis was 9.7 ± 2.6 g/dL, with no significant difference between the groups (*p* = 0.404). Risk stratification by Sokal and ELTS scores did not differ significantly between the groups (Sokal *p* = 0.086; ELTS *p* = 0.176), although a numerically higher proportion of high-risk patients was observed in the generic group.

Transcript-type data were available for 60 patients. The most common BCR::ABL1 transcript type was e14a2 (61.7%), followed by e13a2 (35.0%). Rare transcript variants were found in two patients, both from the generic group: one with the rare e14a3 transcript and the other with an atypical fusion transcript not corresponding to any known common fusion types. No significant differences in transcript distribution were observed between treatment groups (*p* = 0.103)

Additional cytogenetic abnormalities (ACAs) were identified in nine patients (12.7%), with a higher proportion observed in the generic imatinib group than in the original group (20.0% vs. 8.7%, *p* = 0.160). Among these, major route abnormalities were found in four patients (5.6%), each involving either +8, +Ph, i(17), or −7. There was no significant difference between the groups (4.0% vs. 6.5%, *p* = 0.559). Although not statistically significant, the presence of ACAs was numerically more frequent in the generic group, while the proportion of major route abnormalities was similar.

Regarding healthcare coverage, the majority of patients were enrolled under the UCS (69%), followed by the SSS (19.7%) and CSMBS (11.3%), with no significant difference between groups (*p* = 0.686). The median follow-up duration was significantly longer in the original imatinib group (97.9 months, IQR 81.7–115.4) than in the generic group (44.6 months, IQR 29.9–54.5; *p* < 0.01), reflecting the later introduction of generic imatinib into clinical practice. The baseline characteristics of the participants are summarized in Table 1.

### 3.2. Treatment Response and Depth of Response

At 3 months, CHR was achieved in 97.8% of patients in the original imatinib group and 96.0% in the generic group (*p* > 0.999). The cumulative rates of CCyR at 3, 6, and 12 months in the original versus generic groups were 23.9% vs. 32.0%, 50.0% vs. 48.0%, and 73.3% vs. 70.8%, respectively (*p*-values: 0.462, 0.872, 0.825). The cumulative MMR rates at the same time points were 0% vs. 4.0%, 10.9% vs. 8.0%, and 35.6% vs. 41.7%, respectively (*p*-value: 0.352, >0.999, 0.618).

Although pointwise comparisons at individual time points were not statistically significant, a mixed-effects model revealed a statistically significant group-by-time interaction for both CCyR (*p* < 0.001) and MMR (*p* < 0.001). This suggests that the trajectories of cytogenetic and molecular responses differed between treatment groups, with original imatinib achieving a more favorable trend in response depth over time.

**Figure 1:**
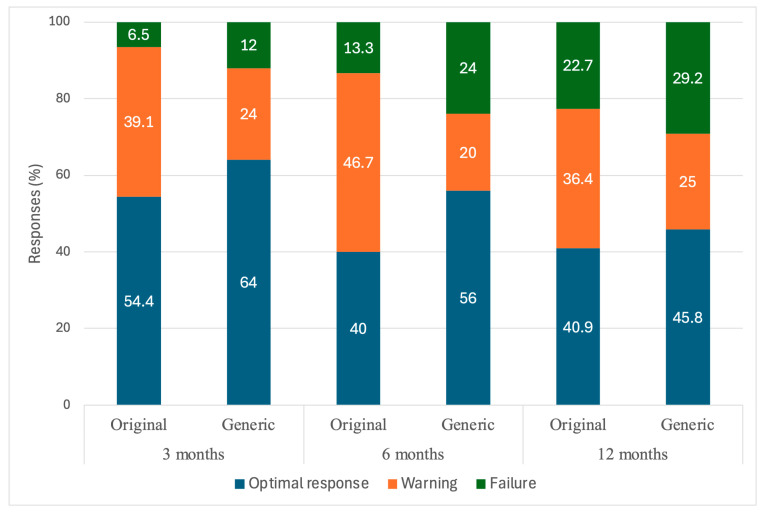
Response rates and depth of response in patients treated with the original and generic imatinib at 3, 6, and 12 months.

The distribution of ELN response categories (optimal, warning, and failure) was comparable between the groups at all milestones. At 12 months, an optimal response was observed in 40.9% of patients in the original group and 45.8% in the generic group (*p* = 0.617), while failure rates were 22.7% and 29.2%, respectively. A detailed summary of the treatment responses is presented in Table 2 and Figure 1.

At 12 months, 18 patients (25.4%) were switched to second-generation TKIs. Of these, 17 patients (94.4%) switched due to treatment failure, and one patient switched due to intolerance. After the 12-month mark, three additional patients experienced treatment failure with frontline imatinib and were subsequently switched to second-line therapy. Nilotinib was the most used second-line TKI, prescribed in 20 patients, while one patient received dasatinib directly as second-line therapy. Among those treated with nilotinib, seven patients experienced further failure and were subsequently switched to dasatinib as a third-line therapy. Notably, three patients developed resistance to three TKIs: imatinib, nilotinib, and dasatinib.

The proportion of patients requiring TKI switching at 3, 6, and 12 months was not significantly different between the original and generic imatinib groups at each individual timepoint. However, a repeated-measures mixed-effects model revealed a statistically significant difference in switching patterns over time between the two groups (*p* = 0.009).

### 3.3. Event-Free and Overall Survival

**Figure 2:**
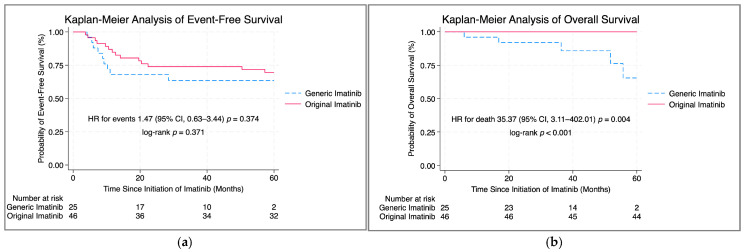
Kaplan−Meier survival curves comparing outcomes in patients treated with original versus generic imatinib as frontline treatment. (**a**) Event-free survival (EFS); (**b**) Overall survival (OS).

After a median follow-up of 80.3 months, there was no statistically significant difference in the EFS between patients treated with original versus generic imatinib (log-rank *p* > 0.05, Figure 2a). The estimated 1-year EFS was 84.8% in the original group and 68.0% in the generic group. At 5 years, the EFS was 69.6% versus 63.5%, respectively. Univariable analysis identified delayed achievement of CCyR beyond 12 months as a significant predictor of inferior EFS (HR 9.36, 95% CI 3.63–24.1, *p* < 0.001). In contrast, OS was significantly better in the original imatinib group. The 5-year OS was 95.6% for original imatinib compared with 65.4% for generic imatinib (log-rank *p* < 0.001; Figure 2b). The univariable analysis of factors associated with disease-related events and overall mortality is shown in Table 3.

Multivariable Cox regression analysis was performed, incorporating age at diagnosis, ELTS score, presence of ACAs, and use of generic imatinib. The analysis showed that older age (HR 1.06, 95% CI 0.99–1.13, *p* = 0.064), intermediate ELTS risk (HR 13.58, 95% CI 0.78–234.84, *p* = 0.073), presence of ACAs at diagnosis (HR 10.45, 95% CI 1.21–90.58, *p* = 0.033), and use of generic imatinib (HR 422.12, 95% CI 3.22–55,304.32, *p* = 0.015) were associated with an increased risk of mortality. Among these, only the presence of ACAs and the use of generic imatinib were statistically significant and independently associated with overall survival.

To address the imbalance in follow-up duration between the treatment groups, a time-dependent Cox regression analysis was performed, modeling treatment exposure (original vs. generic imatinib) as a time-varying covariate. The use of generic imatinib remained significantly associated with inferior overall survival in this model.

### 3.4. Adverse Events

Overall, the adverse event rates were comparable between the original and generic imatinib groups. Anemia was significantly more frequent in the generic group (28.0% vs. 8.7%; *p* = 0.043), whereas muscle cramps occurred exclusively in the original group (15.2% vs. 0%; *p* = 0.047). No significant differences were observed in the incidence of thrombocytopenia, leukopenia, nausea, rash, or the need for dose adjustment due to treatment-related toxicity between the two groups. A detailed summary of adverse events is presented in Table 4.

## 4. Discussion

This study compared the clinical efficacy and safety outcomes of generic and original imatinib in patients with chronic-phase CML treated at a tertiary center in Thailand. Our findings demonstrate comparable early hematologic and cytogenetic responses but significantly inferior OS in the generic imatinib group, consistent with emerging concerns from some international real-world studies.

### 4.1. Baseline Characteristics

While most baseline characteristics were balanced between groups, patients receiving generic imatinib were older and had a higher proportion with ECOG performance status of 1, although neither difference reached statistical significance. The lower mean hemoglobin level of 9.7 g/dL across the cohort may reflect contributing factors. Delayed diagnosis and advanced disease burden at presentation, common in resource-limited settings, may have contributed to anemia, irrespective of sex. Furthermore, approximately one-third of the Thai population are carriers of thalassemia or have mild forms of thalassemia disease, which can lower baseline hemoglobin levels [20].

Although the Sokal and ELTS risk classifications were evenly distributed, we observed an unexpected finding: intermediate-risk ELTS scores were independently associated with worse OS, more than high-risk scores. This paradox may be due to the small sample size, number of events in the high-risk group, misclassification, or residual confounding factors. Additionally, patients with intermediate risk may have unmeasured adverse factors that are not captured by standard scoring systems.

BCR::ABL1 transcript type distribution did not differ significantly between the groups; however, rare transcripts were noted in the generic imatinib group (one e14a3 and one atypical variant). While both patients achieved CCyR without complications, these rare variants can complicate molecular monitoring and may have unclear prognostic implications.

In this study, ACAs were found in 12.7% of the cohort, while major route abnormalities (+8, +Ph, i(17q), −7) were found in 5.6%, consistent with the prevalence in previous reports [21,22,23]. Although not statistically significant, ACAs and major route abnormalities were slightly more frequent in the generic group, which may indicate an increased risk of disease instability. Their prognostic significance has been emphasized in the ELN and WHO guidelines, especially when new clonal evolution occurs during therapy or in association with poor treatment response [18,21,24,25].

Regarding healthcare schemes, most patients in the generic group were covered under the SSS or UCS, whereas those in the original group were more likely to be covered under the CSMBS. Although no significant differences in response or toxicity were noted by scheme, the systemic distinction driven by national policy could introduce unmeasured disparities in access to follow-up care, adherence, and continuity of treatment.

A key limitation in baseline comparability is the significantly shorter median follow-up duration in the generic group (45 vs. 98 months), which reflects the policy-driven entry of generic imatinib into the Thai market in 2017. To address this imbalance, treatment exposure was modeled as a time-varying covariate in a time-dependent Cox regression analysis. This approach helps mitigate potential bias arising from differences in follow-up duration. Notably, the survival disadvantage associated with the generic imatinib group remained statistically significant in this model, suggesting that the observed difference in OS is unlikely to be explained solely by unequal follow-up times.

### 4.2. Response Outcomes

At 3, 6, and 12 months, CCyR and MMR rates were similar between the groups. However, using a mixed-effects model for repeated measures, a significant group-by-time interaction was detected for both CCyR and MMR, indicating that patients receiving original imatinib achieved deeper responses more consistently over time. These findings suggest that while early response milestones may appear similar, response durability and trajectory may differ.

Although the proportion of patients requiring a switch to second-generation TKIs did not differ significantly between groups at individual time points, a mixed-effects model revealed a statistically significant difference in switching patterns over time (*p* = 0.009). This suggests that patients receiving generic imatinib may experience a different response trajectory with higher cumulative switching rates over time. Such longitudinal treatment dynamics may not be captured in cross-sectional comparisons and warrant further investigation.

### 4.3. Event-Free Survival (EFS) and Overall Survival (OS)

EFS did not differ significantly between the groups. In contrast, OS was significantly shorter in the generic group. These findings are consistent with real-world reports showing increased rates of treatment failure over time and a higher likelihood of treatment discontinuation with generic imatinib, attributed to intolerance or lower treatment persistence [15,16]. These issues may compromise long-term disease control and ultimately affect survival. While the number of deaths was small, possible contributors may include disease progression (e.g., sudden blast crisis), comorbidities, or non-CML-related causes.

Importantly, while baseline characteristics were not significantly different, some imbalanced findings may have affected the outcomes, particularly ACAs and major route abnormalities, which have been associated with poorer responses to TKIs and an increased risk of disease progression, diminishing survival outcomes [18,21,24,25]. In our study, patients in the generic group showed a slightly higher rate of ACAs and shorter follow-up, both of which may have contributed to the poorer OS. Further investigation into the cumulative impact of cytogenetic complexity and treatment settings on long-term outcomes is warranted.

The lack of access to consistent drug formulations in generic groups due to central procurement and periodic brand switching may also contribute to long-term survival differences.

### 4.4. Limitations

This study has several limitations. First, its retrospective design and modest sample size introduce the potential for selection bias, misclassification, and incomplete data, limiting the strength of causal inference. Although we adjusted for key covariates and applied a time-dependent Cox regression model to account for the follow-up duration, residual confounding remains possible. Second, we lacked comprehensive data on comorbidities or underlying health conditions, which may have influenced treatment tolerance, treatment choices, and long-term survival. This limitation also restricted our ability to distinguish CML-related mortality from deaths due to non-CML-related causes.

Third, treatment adherence and socioeconomic factors, both critical determinants of real-world effectiveness, were not systematically recorded in the medical records. Adherence may have been affected by factors such as limited patient education, financial constraints, or changes in generic drug formulations due to centralized procurement processes. In addition, frequent changes in the generic imatinib-typically every 6 to 12 months due to national procurement cycles-and variability in brand allocation across healthcare schemes prevent us from performing brand-specific analyses to evaluate the potential impact of each formulation on clinical outcomes. Furthermore, brand switching over time may have introduced variability in drug bioavailability, which could have influenced treatment efficacy or tolerability.

While rare BCR::ABL1 transcript variants were identified in the generic group, the small number of cases limits the interpretation of their clinical significance. Lastly, our modest sample size, especially within subgroups such as high-risk ELTS classification, reduces the statistical power to detect subtle differences and may have contributed to the unexpected survival trends observed.

### 4.5. Future Directions

Prospective studies with standardized follow-up durations, detailed comorbidity data, and stratification by generic brand are needed to better understand the long-term outcomes. The inclusion of pharmacokinetic assessments and patient-reported outcomes may help clarify the impact of formulation differences on efficacy, tolerability, and survival.

## 5. Conclusions

In this real-world cohort, generic imatinib demonstrated comparable early treatment efficacy and EFS to that of original imatinib. However, patients receiving original imatinib had significantly better OS, indicating the need for further evaluation of the factors influencing long-term outcomes. Mixed-effects analysis revealed significant differences in the trajectory of cytogenetic and molecular responses, favoring the original imatinib. While adverse events were generally manageable in both groups, certain toxicities differed by formulation. These findings underscore the importance of long-term monitoring and further research on survival-influencing factors. Early achievement of cytogenetic and molecular responses remains a strong predictor of favorable outcomes, regardless of treatment formulation. Generic imatinib remains a viable treatment option in settings where cost is a major consideration; however, closer follow-up may be necessary to ensure optimal long-term disease control and survival.

## Figures and Tables

**Table 1:** Baseline characteristics of patients receiving original versus generic imatinib.

Baseline Characteristics	Total (*n* = 71)	Original Imatinib (*n* = 46)	Generic Imatinib (*n* = 25)	*p*-Value
Sex, *n* (%) Male Female	39 (54.9) 32 (45.1)	25 (54.4) 21 (45.6)	14 (56.0) 11 (44.0)	0.894
Age, years (median (IQR))	40 (33–54)	38.5 (33–49)	50 (33–66)	0.061
ECOG Performance Status, *n* (%) 0 1	46 (64.8) 25 (35.2)	33 (71.7) 13 (28.3)	13 (52.0) 12 (48.0)	0.096
Hemoglobin at diagnosis, g/dL (mean ± SD)	9.7 ± 2.6	9.9 ± 2.5	9.3 ± 2.6	0.404
Initial white blood cell count, ×10^9^/L (median (IQR))	165.1 (90.4–336.1)	137.4 (90.4–284.4)	209.5 (105.7–350.2)	0.354
Platelet, ×10^9^/L (median (IQR))	527 (257–784)	542 (268–997)	495 (257–650)	0.204
Sokal Score, *n* (%) Low Intermediate High	20 (28.2) 17 (23.9) 34 (47.9)	17 (37.0) 10 (21.7) 19 (41.3)	3 (12.0) 7 (28.0) 15 (60.0)	0.086
ELTS Score, *n* (%) Low Intermediate High	30 (42.2) 20 (28.2) 21 (29.6)	23 (50.0) 12 (26.1) 11 (23.9)	7 (28.0) 8 (32.0) 10 (40.0)	0.176
BCR::ABL1 fusion gene (*n* = 60) e13a2 (b2a2) e14a2 (b3a2) Others	21 (35.0) 37 (61.7) 2 (3.3)	16 (41.0) 23 (59.0) 0 (0.0)	5 (23.8) 14 (66.7) 2 (9.5)	0.103
ACA, *n* (%) Major route abnormality, *n* (%)	9 (12.7) 4 (5.6)	4 (8.7) 3 (6.5)	5 (20.0) 1 (4.0)	0.160 0.559
Healthcare Scheme, *n* (%) CSMBS UCS SSS	8 (11.3) 49 (69.0) 14 (19.7)	6 (13.1) 30 (65.2) 10 (21.7)	2 (8.0) 19 (76.0) 4 (16.0)	0.686
Follow-up Period, months (median (IQR))	80.3 (52.0–106.4)	97.9 (81.7–115.4)	44.6 (29.9–54.5)	<0.001

**Table 2:** Treatment responses and depth of response at 3, 6, and 12 months.

Outcome Category	Timepoint	Original Imatinib (*n* = 46)	Generic Imatinib (*n* = 25)	*p*-Value Compare at Each Timepoint	Repeated Measure *p*-Value
CHR, *n* (%)	3 months	45 (97.8)	24 (96.0)	>0.999	0.487
	6 months	46 (100.0)	25 (100.0)	>0.999	
	12 months	45 (100.0)	25 (100.0)	>0.999	
CCyR, *n* (%)	3 months	11 (23.9)	8 (32.0)	0.462	<0.001
	6 months	23 (50.0)	12 (48.0)	0.872	
	12 months	33 (73.3)	17 (70.8)	0.825	
MMR, *n* (%)	3 months	0 (0.0)	1 (4.0)	0.352	<0.001
	6 months	5 (10.9)	2 (8.0)	>0.999	
	12 months	16 (35.6)	10 (41.7)	0.618	
ELN 2013 Response Outcomes, *n* (%)					
- Optimal - Warning - Failure	3 months	25 (54.4)	16 (64.0)	0.352	0.249
		18 (39.1)	6 (24.0)		
		3 (6.5)	3 (12.0)		
- Optimal - Warning - Failure	6 months	18 (40.0)	14 (56.0)	0.080	
		21 (46.7)	5 (20.0)		
		6 (13.3)	6 (24.0)		
- Optimal - Warning - Failure	12 months	18 (40.9)	11 (45.8)	0.617	
		16 (36.4)	6 (25.0)		
		10 (22.7)	7 (29.2)		
Switched to 2nd generation TKI, *n* (%)	3 months	2 (4.4)	1 (4.0)	>0.999	0.009
	6 months	7 (15.0)	7 (28.0)	0.196	
	12 months	11 (24.4)	7 (29.2)	0.670	

**Table 3:** Univariable analysis of factors associated with disease-related events and overall mortality in patients with CML receiving frontline imatinib.

Variables	Total *n* = 71	Total Event *n* = 23	Event, *n* (%)			Total Death *n* = 7	Death, *n* (%)		
			HR	95% CI	*p*-Value		HR	95% CI	*p*-Value
Sex Male Female	39 32	12 (30.8) 11 (34.4)	Ref 1.09	0.48–2.47	0.834	3 (7.5) 4 (12.5)	Ref 1.95	0.43–8.89	0.390
Age at diagnosis, years (median (IQR))	40 (33–54)	47 (30–57)	1.02	0.99–1.05	0.190	49 (33–75)	1.07	1.01–1.13	0.019
ECOG 0 1	46 25	13 (28.3) 10 (40.0)	Ref 1.76	0.77–4.03	0.178	3 (6.5) 4 (16.0)	Ref 3.44	0.75–15.73	0.110
Sokal Score Low Intermediate High	20 17 34	7 (35.0) 7 (41.2) 9 (26.5)	Ref 1.35 0.78	0.47–3.85 0.29–2.09	0.577 0.620	1 (5.0) 3 (17.7) 3 (8.8)	Ref 4.09 2.16	0.42–39.66 0.22–20.83	0.224 0.505
ELTS Score Low Intermediate High	30 20 21	9 (30.0) 8 (40.0) 6 (28.6)	Ref 1.57 0.86	0.61–4.09 0.36–2.84	0.351 0.986	1 (3.3) 4 (20.0) 2 (9.5)	Ref 6.38 3.43	0.71–57.71 0.31–37.94	0.099 0.314
ACAs Major route abnormality	9 (12.7) 4 (5.6)	7 (30.4) 2 (8.7)	7.13 2.14	2.87–17.74 0.50–9.18	<0.001 0.304	4 (57.1) 2 (28.6)	11.92 6.49	2.65–53.68 1.25–33.63	0.001 0.026
Healthcare scheme CSMBS UCS SSS	8 49 14	2 (25.0) 17 (34.7) 4 (28.6)	Ref 1.44 1.18	0.33–6.22 0.22–6.42	0.629 0.852	1 (12.5) 5 (10.2) 1 (7.1)	Ref 0.66 0.46	0.07–5.80 0.03–7.60	0.706 0.590
Imatinib Treatment Original Generic	46 25	14 (30.4) 9 (36.0)	Ref 1.47	0.63–3.44	0.374	2 (4.4) 5 (20.0)	Ref 35.37	3.11–402.01	0.004
Time to achieve CCyR ≤12 months >12 months	48 23	6 (12.5) 17 (73.9)	Ref 9.36	3.63–24.1	<0.001	1 (2.1) 6 (26.1)	Ref 12.99	1.56–108.05	0.018
Time to achieve MMR ≤12 months >12 months	16 55	2 (12.5) 21 (38.2)	Ref 3.54	0.83–15.1	0.088	1 (6.3) 6 (10.9)	Ref 1.60	0.19–13.34	0.662

**Table 4:** Comparison of adverse events between original and generic imatinib.

Adverse Event	Total (*n* = 71)	Original Imatinib (*n* = 46)	Generic Imatinib (*n* = 25)	*p*-Value
Non-hematologic events				
Muscle cramp	7 (9.9%)	7 (15.2)	0 (0.0)	0.047
Edema	10 (14.1%)	5 (10.9)	5 (20.0)	0.307
Nausea	8 (11.3%)	7 (15.2)	1 (4.0)	0.246
Rash	3 (4.2%)	2 (4.4)	1 (4.0)	>0.999
Hematologic events				
Anemia	11 (15.5)	4 (8.7)	7 (28.0)	0.043
Thrombocytopenia	18 (25.4)	10 (21.7)	8 (32.0)	0.342
Leukopenia	18 (25.4)	9 (19.6)	9 (36.0)	0.128

## Data Availability

The data presented in this study are available on reasonable request from the corresponding author. The data are not publicly available due to privacy and ethical restrictions.

## Abbreviations

The following abbreviations are used in this manuscript:

ACAs	Additional cytogenetic abnormalities
AEs	Adverse events
AP	Accelerated phase
BP	Blastic phase
CCyR	Complete cytogenetic response
CHR	Complete hematologic response
CI	Confidence interval
CML	Chronic myeloid leukemia
CSMBS	Civil Servant Medical Benefit Scheme
ECOG	Eastern Cooperative Oncology Group
EFS	Event-free survival
ELN	European LeukemiaNet
ELTS	EUTOS Long-Term Survival
FISH	Fluorescence in situ hybridization
GIST	Gastrointestinal stromal tumor
HR	Hazard ratio
IQR	Interquartile range
MMR	Major molecular response
OS	Overall survival
PCR	Polymerase chain reaction
Ph	Philadelphia chromosome
SD	Standard deviation
SSS	Social Security Scheme
TKI	Tyrosine kinase inhibitor
UCS	Universal Coverage Scheme
WBC	White blood cell
